# Treatment with Huisheng oral solution inhibits the development of pulmonary thromboembolism and metastasis in mice with Lewis lung carcinoma

**DOI:** 10.3892/ol.2013.1661

**Published:** 2013-11-06

**Authors:** WEI WANG, HONG WANG, CHUN-MEI WANG, SI GOU, ZHONG-HUA CHEN, JIE GUO

**Affiliations:** 1 Department of Pharmacology, West China School of Pharmacy, Sichuan University, Chengdu, Sichuan 610041, P.R. China; 2 Institute of Blood Transfusion, Chinese Academy of Medical Sciences & Peking Union Medical College, Chengdu, Sichuan 610081, P.R. China; 3 Key laboratory of Drug Targeting, Ministry of Education, West China School of Pharmacy, Sichuan University, Sichuan 610041, P.R. China; 4 Chengdu Diao Tianfu Pharmaceutical Group Co., Ltd., Chengdu, Sichuan 610041, P.R. China

**Keywords:** Huisheng oral solution, Lewis lung carcinoma, hypercoagulable, pulmonary thrombus, metastasis

## Abstract

The aim of this study was to investigate whether Huisheng oral solution (HSOS) has an inhibitory effect on the development of pulmonary thrombosis and metastasis in mice with Lewis lung carcinoma (LLC), and to explore the possible mechanisms involved. A mouse model of LLC was developed, and model mice were divided into either a treatment group or a control group to undergo treatment with HSOS or normal saline. Normal mice treated with saline were used as normal controls. On day 25 after treatment, blood samples were drawn from the eyes of half the mice in each group to determine blood cell counts and plasma levels of D-Dimer and vascular endothelial growth factor (VEGF), while heart blood samples were collected from the remaining mice to measure the rate of thrombin-induced platelet aggregation. For all mice, pathological analyses of the cerebrum, lung, mesentery, femoral vein, external iliac vein and spleen were performed. Tumors were weighed to assess the impact of HSOS treatment on tumor growth, and the number of thrombi, metastatic nodules and neovessels in the tumor tissue were counted. In addition, 24 normal New Zealand rabbits were divided into two groups and treated with either HSOS or normal saline to determine the rates of ADP-, collagen- or thrombin-induced platelet aggregation. Compared with the model group, HSOS treatment decreased the incidence of pulmonary thromboembolism and metastasis, the number of metastatic nodules, the plasma levels of D-dimer and VEGF, the rate of collagen-induced platelet aggregation in rabbits and the numbers of leukocytes and tumor neovessels (P<0.05 for all). It increased the thymus and spleen coefficients and the number of platelets (P<0.05 for all), but had no significant effect on thrombin-induced platelet aggregation in mice and rabbits, ADP-induced platelet aggregation in rabbits, or the number of red blood cells. The reduced rate of tumor growth was 9.7% in mice treated with HSOS. HSOS treatment effectively reduced the development of pulmonary thromboembolism and metastasis in mice bearing LLC via mechanisms possibly associated with ameliorating a blood hypercoagulable state, decreasing tumor angiogenesis and enhancing immunity.

## Introduction

Metastasis is the predominant cause of death in cancer patients, and the majority of patients with malignant tumors will develop varying degrees of metastasis by the time they reach the late stage of the disease (1–5). As metastasis is the leading cause of treatment failure and tumor relapse, the inhibition of tumor metastasis is a key element of anticancer treatment. Numerous patients with malignant tumors are in a hypercoagulable state, which can cause various types of thromboembolism. The latest data show that 9% of cancer patients die of thromboembolism. Venous thromboembolism (VTE) is one of the major complications of malignant tumors, with an incidence of 4–6% (6–9). VTE is second only to the malignancy itself as a cause of mortality in cancer patients (10–11). Studies have shown that a hypercoagulable state is closely associated with the metastasis of malignant tumors (12). The present study aimed to investigate whether Huisheng oral solution (HSOS) inhibits tumor metastasis by improving the hypercoagulable state in patients with malignancies.

HSOS was developed based on a traditional recipe, known as Huazheng Huisheng Dan, which was recorded in a medical book from the Qing Dynasty. After 17 years of clinical use, HSOS as an adjuvant chemotherapy agent has been demonstrated to significantly improve the quality of life and survival of cancer patients (13–15). In view of the correlation between the clinical efficacy of HSOS in malignant tumors and blood stasis syndrome, and the correlation between blood stasis syndrome and the hypercoagulable state and thromboembolism, we hypothesized that promoting blood circulation and removing blood stasis may inhibit the metastasis of malignant tumors based on traditional Chinese medicine theory (16). By developing a middle- to old-aged C57 mouse model of Lewis lung carcinoma (LLC) with concurrent thromboembolism, we investigated the effect of HSOS treatment on the hypercoagulable state, assessed its role in preventing tumor metastasis through promoting blood circulation and removing blood stasis, and explored the possible mechanisms involved. The results obtained may provide convincing experimental evidence to support the notion that Chinese medicine plays an active role in the inhibition of tumor metastasis.

## Materials and methods

### Materials and equipment

#### Animals and tumor cell lines

Eight- to twelve-month-old female C57BL/6 mice of specific pathogen-free grade, weighing between 27 and 30 g, and male New Zealand rabbits, weighing between 2 and 2.5 kg, were provided by the Huaxi Laboratory Animal Center of Sichuan University [certificate nos. SCXK (chuan) 2008–09 and SCXK (chuan) 2008–10; Chengdu, China]. The animals were allowed to adapt to their respective environment and food for six to seven days prior to the start of the experiments. The LLC cell line was obtained from the Laboratory of Tumor Biology at West China Hospital of Sichuan University (Chengdu, China). The study was approved by the Ethical Review Committee of the Society for Laboratory Automation and Screening (SLAS, Chengdu, China), and all animal procedures were approved by the Institutional Animal Care and Use Committee of the Society for Laboratory Automation and Screening.

#### Drugs and reagents

HSOS (20110203; Chengdu Tianfu Pharmaceutical, Chengdu, China), trypsin (Amresco, Solon, OH, USA), fetal bovine serum (Gibco, Carlsbad, CA, USA), Dulbecco’s modified Eagle’s medium (DMEM; Gibco), ADP (Chrono-log Corporation, Havertown, PA, USA), collagen (Chrono-log Corporation) and thrombin (384–386; Chrono-log Corporation) were obtained commercially. Platelet washing solution and modified Tyrode’s solution have been described previously (17,18). VEGF and D-dimer ELISA kits (201204; Shanghai Fengxiang Biological Technology Co., Ltd., Shanghai, China), anti-CD34 primary antibody (13012; Santa Cruz Biotechnology, Inc., Santa Cruz, CA, USA), biotinylated goat anti-rabbit IgG (V0527; Beijing Zhongshan Golden Bridge Biotechnology Co., Ltd., Beijing, China), the DAB chromogenic kit (753223A; Beijing Zhongshan Golden Bridge Biotechnology Co., Ltd.), sodium citrate and EDTA-2Na were also obtained commercially.

#### Main equipment

An MLR-351 CO_2_ incubator (Sanyo, Osaka, Japan), CKX41 inverted microscope (Olympus, Tokyo, Japan), 81m-25 optical microscope (Olympus), CELL-DYN1700 blood cell analyzer (Abbott Diagnostics, Abbott Park, IL, USA), 560CA platelet aggregation detector (Chrono-log Corporation), MK3 microplate reader (Thermo Electron, Waltham, MA, USA), KEN-006 high-speed centrifuge (Kendro Instruments, Newtown, CT, USA), clean bench (Wujiang Huazhao Purifying Equipment Co., Ltd., Jiangsu, China) and refrigerator (Revco Technologies, Asheville, NC, USA) were used in this study.

### Methods

#### Cell suspension preparation

LLC cells in logarithmic phase were used in this study. After pouring off the supernatant, adherent cells were digested with 0.25% trypsin, and harvested cells were suspended in 10% DMEM. The suspended cells were then centrifuged at 150 × g for 3 min, re-suspended with DMEM and counted to adjust the density of cells to 1×10^7^ cells/ml.

#### Tumor cell injection

Tumor cell suspension (0.2 ml per mouse) was subcutaneously injected into the left armpit of female C57BL/6 mice.

#### Animal groups and treatments

The mice injected with tumor cell suspension were divided into two groups: a model group (n=50) and a treatment group (n=50). Fifty normal mice were used as normal controls. The treatment group was intragastrically administered with HSOS at 0.25 ml·d^−1^ (~16.7-fold the human dose) for 25 consecutive days, while the normal control group and model group were administered equal volumes of normal saline. Twenty-four New Zealand rabbits were divided into either a treatment group or a normal control group (n=12 for each group), and both groups were intragastrically administered HSOS at 5 ml·kg^−1^·d^−1^ (~10-fold the human dose). The doses used in this study were determined based on a pilot study.

#### Preparation of platelet-rich plasma (PRP) and washed platelets

Blood samples collected in tubes containing 3.8% sodium citrate [blood samples were mixed with sodium citrate at a ratio of 1:9 (vol/vol)] were centrifuged at 100 × g for 5 min, resulting in a PRP supernatant. The pellet was further centrifuged at 1,200 × g for 10 min, resulting in a platelet-poor plasma (PPP) supernatant. The number of platelets in the resulting PRP was adjusted to 400–500×10^9^/l with the resulting PPP for the determination of ADP- and collagen-induced platelet aggregation. The PRP was centrifuged at 400 × g for 8 min, and the supernatant was decanted. The resulting platelet pellet was washed twice with platelet washing solution and re-suspended in modified Tyrode’s solution. The number of platelets was adjusted to 400–500×10^9^/l for the determination of the rate of thrombin-induced platelet aggregation.

### Measurements

#### Blood cell counts

One hour after drug administration on day 25, blood samples were drawn from the eyes of 25 mice in each group (~0.7 ml per mouse) and placed in tubes containing 10% EDTA-2Na (yielding a final concentration of 4%). Blood samples (0.25 ml) were used for counting blood cells (mainly platelets, leukocytes and red blood cells).

#### Plasma D-dimer and VEGF levels

The remaining blood samples were centrifuged at 900 × g for 20 min, and the supernatants were collected for the measurement of plasma levels of D-dimer and VEGF levels using commercial kits according to the manufacturers’ instructions.

#### Rate of platelet aggregation

One hour after drug administration on day 25, heart blood samples were collected from the remaining mice in each group (~1 ml per mouse) and used to prepare washed platelets. Modified Tyrode’s solution was used as a blank control for the determination of the rate of thrombin-induced platelet aggregation. Subsequently, 10 μl of thrombin (100 U·ml^−1^) was added to 500 μl of washed platelets, and the maximum rate of platelet aggregation after 5 min was measured.

One hour after drug administration on day 10, blood samples were obtained from an ear artery of New Zealand rabbits with a vacuum blood collection needle (~10 ml per rabbit) to prepare the washed platelets. Following this, 2 μl thrombin (100 U·ml^−1^) was added to 500 μl of washed platelets, and the maximum rate of platelet aggregation after 5 min was measured. In addition, PRP was prepared to determine the rates of ADP- and collagen-induced platelet aggregation. PPP was used a blank control. ADP (10 μl) or collagen (1 μl) was added to 500 μl of the PRP to obtain a final concentration of 5 μM or 2 μg·ml^−1^ to determine the maximum rate of platelet aggregation after 5 min.

#### Number of thrombi or metastatic nodules

Tumor-bearing mice were sacrificed to collect the brain, lungs, mesentery, femoral vein and external iliac vein tissues. The tissues were then fixed in 10% formalin, paraffin-embedded (two wax blocks for coronal sections of the brain, two wax blocks for five lung lobes, and one wax block for the femoral vein, external iliac vein or mesentery), sectioned and subjected to HE staining. The number of thrombi or metastatic nodules was counted under a light microscope. Metastatic nodules were classified into four grades based on their diameter: I, <0.5 mm; II, 0.5–1 mm; III, 1–2 mm; and IV, >2 mm. The total number of metastatic nodules was calculated as follows: (Number of grade I nodules × 1) + (number of grade II nodules × 2) + (number of grade III nodules × 3) + (number of grade IV nodules × 4) (19).

#### Tumor weight and reduced rate of tumor growth

Tumors were removed from the animals and weighed, and the reduced rate of tumor growth (%) was calculated as follows: (Average tumor weight for the model group − average tumor weight for the treatment group/average tumor weight for the model group) × 100%.

#### Thymus and spleen coefficients

The thymus and spleen were removed from the animals and weighed to calculate organ coefficients using the following formula: Organ coefficient = organ weight/body weight excluding tumors. In addition, sections of spleen tissue were prepared to observe histopathological changes.

#### Tumor microvessel density (MVD)

Tumor tissue was fixed in 10% paraformaldehyde, paraffin-embedded and sectioned. The expression of CD34 in tumor tissue was detected using the immunohistochemical SP method with a streptavidin-peroxidase kit (Beijing Zhongshan Golden Bridge Biotechnology Co., Ltd.) according to the manufacturer’s instructions. Stained sections were observed under a microscope, and positive cells were stained brown-yellow or yellow in the cytoplasm. The average optical density was determined using the Image-Pro Plus 6.0 image analysis system (Media Cybernetics, Inc., Rockville, MD, USA).

#### Statistical analysis

All statistical analyses were performed using SPSS 17.0 software (IBM, New York, NY, USA). Numerical data are expressed as the means ± standard deviation. Blood cell counts, plasma levels of D-dimer and VEGF and rates of platelet aggregation were compared using one-way analysis of variance. Homogeneity of variance was tested. The LSD test was used when P>0.05 and Dunnett’s T3 test was used when P<0.05. Tumor weights, the number of metastatic nodules and MVD in tumor tissue were compared using the independent samples t-test. The incidence of thrombosis or pulmonary metastasis was compared using the χ^2^ test. P<0.05 was considered to indicate a statistically significant difference.

## Results

### Effect of HSOS treatment on the incidence of pulmonary thrombosis in mice with LLC

**Figure 1:**
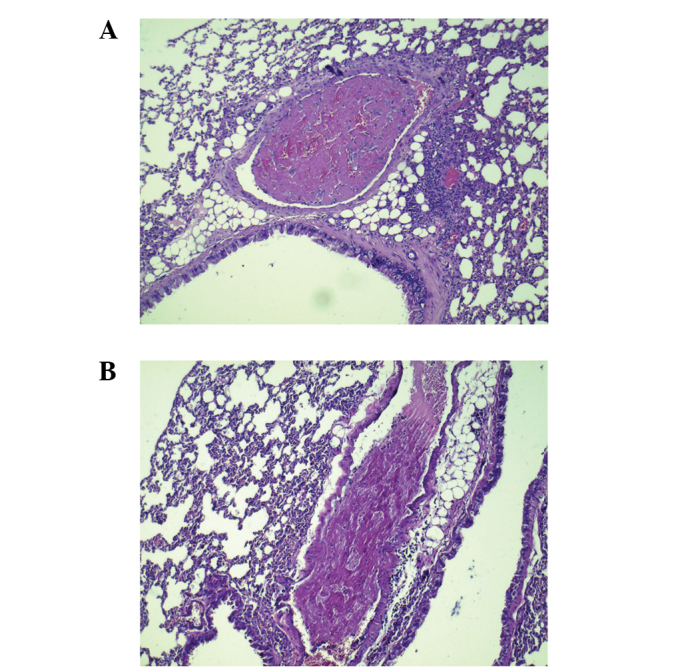
Presence of pulmonary thrombi in mice with Lewis lung carcinoma. (A) Cross section. (B) Longitudinal section. Platelets aggregated and formed trabeculae, which were full of fibrin and red blood cells (hematoxylin and eosin staining; magnification, ×100).

Thrombi were observed only in lung tissues. Compared with the normal control mice, the incidence of pulmonary thrombosis was significantly higher in model mice (0/50 vs. 9/50, P<0.01). HSOS treatment significantly reduced the incidence of pulmonary thrombosis compared with that in the model mice (2/50, P=0.055) (Fig. 1).

### Effect of HSOS treatment on the incidence of pulmonary metastasis in mice with LLC

**Figure 2:**
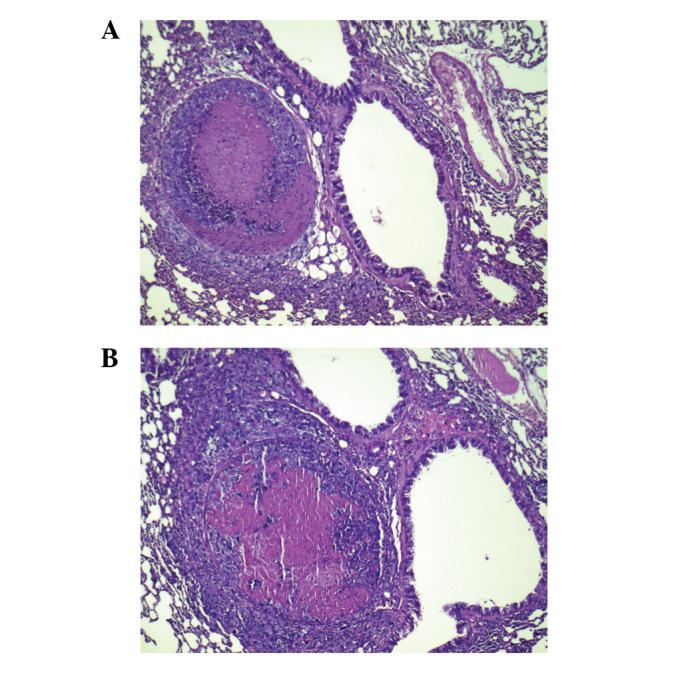
Presence of (A) tumor thrombi and (B) single metastatic nodules in lung tissue of mice with Lewis lung carcinoma. The blood vessel was full of tumor emboli, which clumped together with platelets and resulted in the obstruction of the lumen (hematoxylin and eosin staining; magnification, ×100).

**Figure 3:**
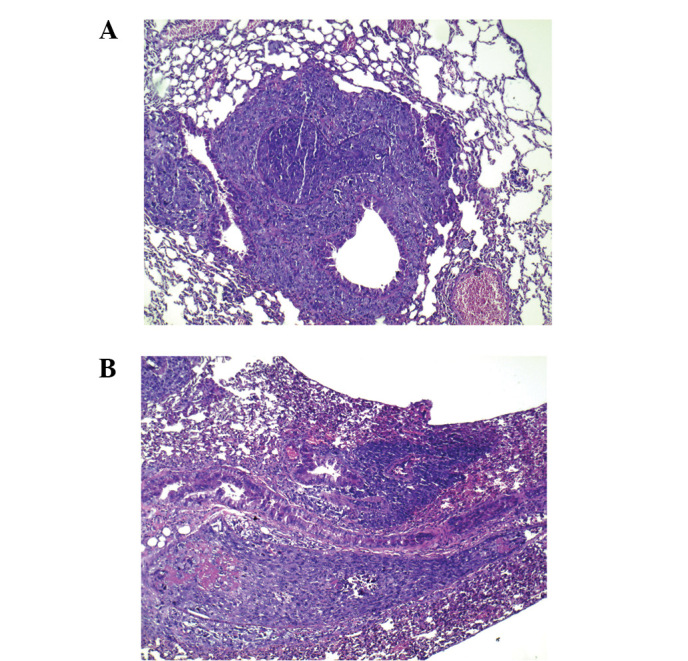
The presence of multiple metastatic nodules in lung tissue of mice with Lewis lung carcinoma. (A) Cross section. (B) Longitudinal section. (Hematoxylin and eosin staining; magnification, ×100).

**Figure 4:**
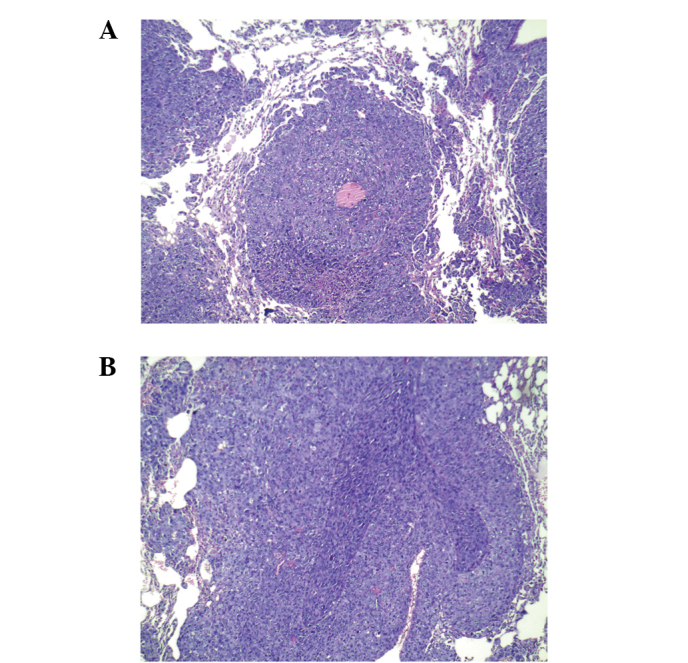
Diffusion of tumor nodules in the entire lung lobe of mice with Lewis lung carcinoma. (A) Cross section. (B) Longitudinal section. Tumor emboli were visible in each metastatic nodule (hematoxylin and eosin staining; magnification, ×100).

Metastatic nodules were observed only in lung tissues. Under the optical microscope, tumor emboli were visible in pulmonary blood vessels. Perivascular tumor nodules were noted and tumor emboli could be observed in each nodule. Tumor nodules were focal or multifocal, and varied in size. Tumor nodules increased in size typically by expansion, with compression of surrounding parenchyma of the lung, or focal invasion into adjacent parenchyma of lung, which destroyed the entire lung lobe (Figs. 2–4). In the model group, the number and size of tumor nodules were large, and diffusion of tumor nodules in the entire lung lobe was observed in certain animals. By contrast, no diffusion of tumor nodules in the entire lung lobe was noted in the treatment group. The average number of metastatic nodules (P=0.031) and the incidence of pulmonary metastasis (44/50 vs. 36/50, P=0.046) were significantly lower in the treatment group than those in the model group.

### Effect of HSOS treatment on tumor growth in mice with LLC

As shown in Table III, the mean tumor weight was marginally lower in the treatment group than in the model group, and the reduced rate of tumor growth was 9.7%.

### Effect of HSOS treatment on blood cell counts and plasma D-dimer levels in mice with LLC

Compared with the normal control group, the white blood cell count and plasma D-dimer levels were significantly higher, and the red blood cell and platelet counts were significantly lower, in the model and treatment groups (P<0.01 for all). However, the white blood cell count (P=0.027) and plasma D-dimer levels (P=0.048) were significantly lower, and the platelet count (P=0.015) was significantly higher, in the treatment group compared with that in the model group, although there was no significant difference in the red blood cell count between the two groups (P=0.77) (Table I).

### Effect of HSOS treatment on the rate of thrombin-, ADP- or collagen-induced platelet aggregation

The rate of thrombin-induced platelet aggregation showed no significant difference among each group in either mice or rabbits. The rate of ADP-induced platelet aggregation also showed no significant difference among each group in rabbits. However, the rate of collagen-induced platelet aggregation was significantly lower in the treatment group than that in the normal control group in rabbits (P<0.01) (Table II).

### Effect of HSOS treatment on plasma VEGF levels and MVD in tumor tissue in mice with LLC

**Figure 5:**
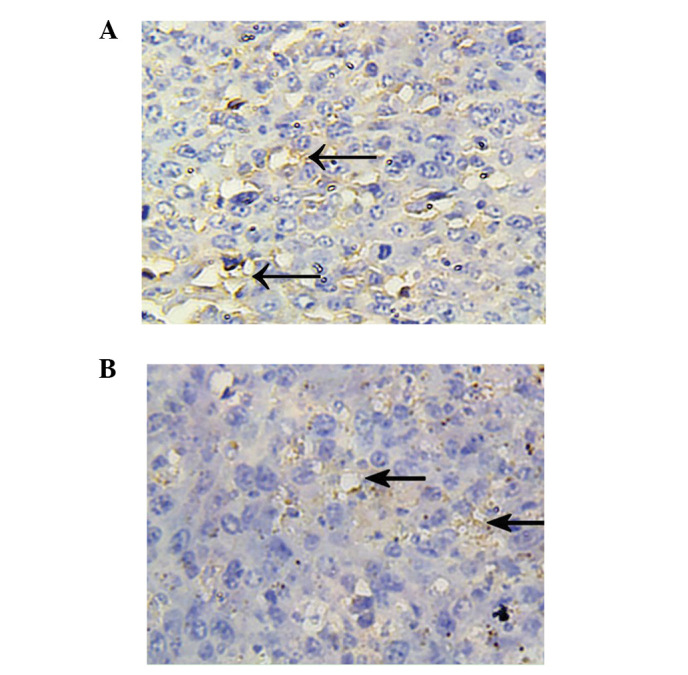
Microvessel density in tumor tissue. (A) Model group. The number of CD34-positive cells was relatively large, and the number of neovessels was higher than that in the treatment group. (B) Treatment group. The number of CD34-positive cells was relatively few, and the number of neovessels was lower than that in the model group. Arrows indicate microvessels. DAB and hematoxylin and eosin staining.

Plasma levels of VEGF were significantly higher in the model and treatment groups than those in the normal control group (P<0.01 for both). However, the plasma level of VEGF in the treatment group was significantly lower than that in the model group (P=0.013) (Table I). The average optical density of MVD in tumor tissue was significantly lower in the treatment group than that in the model group (8061.85±3944.75 vs. 13169.88±9989.43, P<0.01) (Fig. 5).

### Effect of HSOS treatment on immune organs in mice with LLC

**Figure 6:**
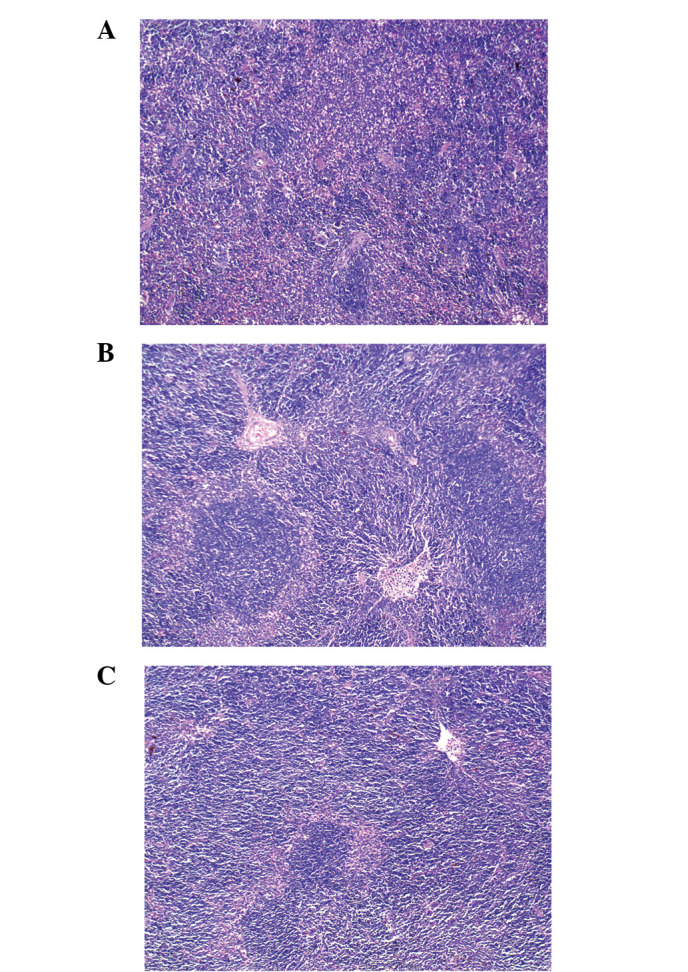
Histology of the spleen. (A) Normal control group. The spleen consisted of the white pulp and red pulp, and the structure of splenic corpuscles was clear. (B) Model group. The number of splenic corpuscles and lymphocytes increased, the splenic cords were widened, and the red pulp was compressed and shrunken. (C) Treatment group. The number of splenic corpuscles and lymphocytes further increased, and the splenic cords were further widened. (Hematoxylin and eosin staining; magnification, ×100).

Compared with the normal control group, the thymus coefficient was significantly lower in the model and treatment groups (P<0.05 for both); however, the thymus coefficient was significantly higher in the treatment group than that in the model group (P=0.045). The spleen coefficient was significantly higher in the model group and treatment group than that in the normal control group (P<0.01 for both); however, the thymus coefficient was significantly higher in the treatment group than that in the model group (P=0.022) (Table III). In both the model and treatment groups, diffuse hyperplasia of spleen lymphocytes was visible, and the red pulp was compressed and shrunken (Fig. 6).

## Discussion

In this study, we selected multiple tissues prone to thromboembolism, including the brain, lung, mesentery, femoral vein and external iliac vein, to observe the occurrence of thrombosis in mice with LLC. Thrombosis associated with LLC metastasis was detected only in the lung. We speculate that this may be due to the fact that LLC cells have a higher affinity for lung tissue. Tumor cells migrate from the injection site with the blood flow to pulmonary blood vessels and form tumor thrombi, which then invade the surrounding lung tissue and result in the occurrence of metastatic nodules (20). When tumor thrombi invade pulmonary blood vessels, vascular endothelial injury occurs. Tumor cells attach to the damaged endothelium and gather into groups to form whirlpools (21). In addition, tumor cells may release a large number of procoagulant substances (22). The presence of vascular endothelial injury, coagulation-promoting substances and hemorheological changes promotes the occurrence of pulmonary vascular thrombosis. In addition, our preliminary results showed a low incidence of pulmonary thromboembolism (1/22) in 6- to 8-week-old C57 mice, which made statistical analysis difficult. For this reason, a relatively large number of middle- to old-aged C57 mice were used in this study in order that the incidence of pulmonary thrombosis (9/50) met the requirements for statistical analysis.

Our observation that treatment with HSOS significantly reduced the incidence of pulmonary thromboembolism and pulmonary metastasis in mice with LLC indicates that HSOS reduces the incidence of pulmonary metastasis by possibly decreasing the incidence of pulmonary thromboembolism. As thrombosis is one of the manifestations of the coagulation states of the body, HSOS may inhibit tumor metastasis by potentially improving the hypercoagulable state of the body.

The finding that treatment with HSOS significantly decreased plasma D-dimer levels in mice with LLC and the rate of collagen-induced platelet aggregation in rabbits indicates that HSOS is capable of improving abnormal coagulation and fibrinolysis caused by malignant tumors and thereby reducing the incidence of pulmonary thromboembolism. This may be one of the mechanisms by which HSOS treatment inhibits cancer metastasis. The increase in D-dimer levels indicates that the generation of thrombin increased, which often leads to thrombosis. The presence of thrombosis enhances fibrinolysis (23), increases blood viscosity and slows down blood flow. Thus, tumor cells may easily attach to the vessel wall and migrate from the blood vessels to form metastatic nodules (24). As HSOS is composed of a variety of substances that may promote blood circulation and remove blood stasis, it may improve blood flow state, decrease blood viscosity, suppress thrombosis, reduce plasma D-dimer levels and indirectly inhibit pulmonary metastasis. Conversely, individual tumor cells in the blood are more likely to be killed by the immune system, while tumor cells aggregated with platelets are able to form tumor thrombi and evade the immune system, resulting in the development of metastasis (25). HSOS treatment may effectively inhibit collagen-induced platelet aggregation, prevent the formation of tumor thrombi and thereby inhibit tumor metastasis.

Neovascularization is essential for tumor proliferation and metastasis. VEGF is the most important angiogenic factor (26). Studies have shown that a hypercoagulable state in patients with malignant tumors promotes tumor angiogenesis. In hypercoagulable states, activated platelets secrete tumor cell growth factors and angiogenic factors, such as platelet-derived growth factor (27), VEGF (28) and angiopoietin-1 (29). VEGF not only promotes the proliferation and division of endothelial cells, and angiogenesis, but also increases vascular permeability, thereby promoting tumor growth and metastasis. Our observation that treatment with HSOS reduced plasma VEGF levels in mice with LLC indicates that HSOS may inhibit tumor angiogenesis. This result is further confirmed by our observation that HSOS reduced MVD in the tumor tissue. Overall, these findings indicate that the improvement of hypercoagulability and the inhibition of tumor angiogenesis is another important mechanism by which HSOS inhibits tumor proliferation and metastasis.

HSOS treatment improved leukocyte and platelet counts in mice with LLC, indicating that HSOS is able to mitigate abnormal peripheral blood cell counts caused by malignant tumors. The increase in the number of leukocytes in mice with LLC may be due to the fact that the tumor cells produced large amounts of inflammatory cytokines to stimulate the release of bone marrow myelocytes and the production of peripheral leukocytes (30). Abnormally increased numbers of leukocytes may gather and adhere to microvessels, and block the blood vessels. In addition, biologically active substances released by leukocytes, such as leukotrienes and prostaglandins, may cause vasoconstriction and platelet aggregation, worsen the hypercoagulable the hyperviscosity state in tumor patients (31), and thereby promote tumor metastasis. Thus, HSOS treatment may reduce leukocyte-induced inflammation and inhibit tumor metastasis. The decrease in red blood cell count and platelet count in mice with LLC may be associated with the consumption of platelets and red blood cells during thrombosis, the destruction of bone marrow hematopoietic function by tumor cells (32) and the excessive consumption of vitamin B12 and folic acid caused by malignant tumors. The observation that HSOS treatment elevated platelet count in mice bearing LLC indicates that HSOS has a protective effect on the body.

Treatment with HSOS may reduce tumor-induced damage to the thymus, strengthen the tumor immune response in the spleen and enhance immune function. Tumor cells secrete immunosuppressive factors and cause thymic atrophy and functional impairment (33). HSOS treatment may alleviate the atrophy of the thymus and therefore exert a protective effect on the thymus. The spleen is the largest peripheral immune organ. Tumor-specific antigens stimulate the spleen to produce an immune response, resulting in the active proliferation of spleen lymphocytes (34). HSOS treatment may further strengthen immune responses in the spleen, indicating that in addition to the tumor immune response, HSOS is involved in enhancing immune function. Enhanced immune function may improve the recognition of tumor antigens and the capacity for killing tumor cells, and reduce pulmonary thrombosis and metastasis. This is another important mechanism underlying the antitumor effect of HSOS.

In conclusion, HSOS is involved in inhibiting tumor metastasis and growth. This inhibitory effect is associated with promoting blood circulation, removing blood stasis and improving hypercoagulability. In the present study, HSOS treatment effectively improved the following hypercoagulability parameters in mice with LLC: D-dimer, platelet aggregation, white blood cell count and the number of pulmonary thrombi. In addition, HSOS treatment inhibits tumor metastasis and growth, possibly by inhibiting tumor angiogenesis and modulating immune function. Our study correlates the anticancer effects of HSOS with the improvement of hypercoagulability, providing a new avenue for the treatment of malignant tumors with Chinese medicine.

In the present study, the reduced rate of tumor growth in aging mice treated with HSOS was 9.7%. We have previously investigated the effect of HSOS treatment on thromboembolism in 6- to 8-week-old mice with LLC and found that the reduced rate of tumor growth was ~40%, which is similar to that reported by others (35). It remains to be investigated whether the lower reduced rate of tumor growth observed in the present study is associated with diminished immune function in aging mice.

## Figures and Tables

**Table I:** Effect of HSOS treatment on blood cell counts and plasma levels of D-dimer and VEGF in mice with LLC (means ± SD, n=25).

	Blood cell count			Plasma	
		
Group	WBC (×10^9^)	RBC (×10^12^)	Platelets (×10^9^)	D-dimer (μg/l)	VEGF (ng/l)
Normal control	5.62±1.76	10.5±0.36	907.3±106.55	46.02±11.81	77.46±48
Model	19.13±5.66^a^	6.61±0.83^a^	343.43±115.84^a^	69.39±17.81^a^	178.7±85.64^a^
Treatment	16.74±3.26^a^,^b^	6.56±0.6^a^	435.87±178.71^a^,^b^	60.72±16.67^a^,^b^	112.26±66.72^b^

HSOS, Huisheng oral solution; VEGF, vascular endothelial growth factor; LLC, Lewis lung carcinoma; WBC, white blood cells; RBC, red blood cells;

a P<0.05 vs. normal control;

b P<0.05 vs. model.

**Table II:** Effect of HSOS treatment on the rates of thrombin-, ADP- and collagen-induced platelet aggregation (means ± SD).

	Rabbits		Mice		
		
	Normal control	Treatment	Normal control	Model	Treatment
Sample size	12	12	10	10	10
Thrombin	96.26±12.32	90±26.89	95.12±27.84	96.50±27.41	95.62±29.05
ADP	52±15	56.4±21.38			
Collagen	101.81±11.37	82.18±9.96^a^			

HSOS, Huisheng oral solution.

a P<0.05 vs. normal control.

**Table III:** Effect of HSOS treatment on immune organs, tumor growth and metastasis (means ± SD, n=50).

Group	Body weight (g)	Tumor weight (g)	Thymus coefficient	Spleen coefficient	Number of metastatic nodules per mouse
Normal control	27.62±3.43	-	0.00179±0.00043	0.0045±0.0021	-
Model	29.24±4.37	5.15±1.17	0.00106±0.0006^a^	0.0129±0.0031^a^	5±4.38
Treatment	28.04±2.9	4.65±0.75	0.0014±0.0008^a^,^b^	0.0154±0.0041^a^,^b^	2.61±4.55^b^

HSOS, Huisheng oral solution.

a P<0.05 vs. normal control;

b P<0.05 vs. model.
